# Power lines, roads, and avian nest survival: effects on predator identity and predation intensity

**DOI:** 10.1002/ece3.1049

**Published:** 2014-03-31

**Authors:** Brett A DeGregorio, Patrick J Weatherhead, Jinelle H Sperry

**Affiliations:** 1 Department of Natural Resources and Environmental Sciences, University of Illinois1102 S. Goodwin Ave, Urbana, Illinois, 61801; 2 Engineer Research and Development CenterChampaign, IL

**Keywords:** Brown-headed cowbirds, edge effects, habitat fragmentation, nest cameras, nest predation, rat snakes

## Abstract

**1** Anthropogenic alteration of landscapes can affect avian nest success by influencing the abundance, distribution, and behavior of predators. Understanding avian nest predation risk necessitates understanding how landscapes affect predator distribution and behavior.

**2** From a sample of 463 nests of 17 songbird species, we evaluated how landscape features (distance to forest edge, unpaved roads, and power lines) influenced daily nest survival. We also used video cameras to identify nest predators at 137 nest predation events and evaluated how landscape features influenced predator identity. Finally, we determined the abundance and distribution of several of the principal predators using surveys and radiotelemetry.

**3** Distance to power lines was the best predictor of predator identity: predation by brown-headed cowbirds (*Molothrus ater*)*,* corvids (*Corvus sp*. and *Cyanocitta cristata*)*,* racers (*Coluber constrictor*), and coachwhips (*Masticophis flagellum*) increased with proximity to power lines, whereas predation by rat snakes (*Elaphe obsoleta*) and raptors decreased. In some cases, predator density may reliably indicate nest predation risk because racers, corvids, and cowbirds frequently used power line right-of-ways.

**4** Of five bird species with enough nests to analyze individually, daily nest survival of only indigo buntings (*Passerina cyanea*) decreased with proximity to power lines, despite predation by most predators at our site being positively associated with power lines. For all nesting species combined, distance to unpaved road was the model that most influenced daily nest survival. This pattern is likely a consequence of rat snakes, the locally dominant nest predator (28% of predation events), rarely using power lines and associated areas. Instead, rat snakes were frequently associated with road edges, indicating that not all edges are functionally similar.

**5** Our results suggest that interactions between predators and landscape features are likely to be specific to both the local predators and landscape. Thus, predicting how anthropogenic changes to landscapes affect nesting birds requires that we know more about how landscape changes affect the behavior of nest predators and which nest predators are locally important.

## Introduction

Anthropogenic habitat alteration can have pervasive effects on wildlife beyond just loss of habitat. The quality of remaining habitat may decline due to an increase in edge habitat or the isolation of remaining patches (Andren 1994). These changes often cause shifts in wildlife species richness, density, or distribution within a landscape (Chalfoun et al. 2002). Installation of linear corridors such as roads and utility right-of-ways may result in relatively little habitat loss, but negatively affect wildlife by creating extensive edge habitat, by inserting early-successional habitat into a forested matrix (Rich et al. 1994), or by modifying the behavior of predators. Linear anthropogenic disruptions can act as travel corridors or barriers for wildlife that can change demographic processes (e.g., increased nest predation, decreased gene flow). Creation of edge habitat associated with linear habitat features can have indirect effects on wildlife by increasing the frequency with which species interact (e.g., nest predation, brood parasitism), often to the detriment of one species (Murcia 1995). Here, we investigate the effect of landscape features (unpaved roads and power line right-of-ways) on avian nest predation in a fragmented landscape and quantify the distribution of the principal nest predators relative to the two features.

The effects of edges on birds have been well studied (Gates and Gysel 1978), and many species have demonstrated sensitivity to factors related to edge at multiple spatial scales (Robinson et al. 1995; Flaspohler et al. 2001). Because nest survival is an important component of songbird demography, edge effects on rates of nest predation for breeding songbirds have frequently been examined (Donovan and Thompson 2001; Manolis et al. 2002). At broad spatial scales, nest predation may increase for forest songbirds as landscapes become more fragmented (Robinson et al. 1995). At finer scales, proximity to edge may negatively influence nest survival within a habitat patch (King and Byers 2002; Manolis et al. 2002) by increasing the risk of nest predation (Lloyd and Martin 2005). In some cases, however, no demonstrable edge effect on songbird nest predation has been found (e.g., Robinson and Wilcove 1994; Hanski et al. 1996), leading researchers to conclude that edge effects may be context specific (Donovan et al. 1997; Lahti 2001). In some cases, edge effects may be species or nesting guild-specific (Flaspohler et al. 2001). Additionally, not all edges may function in the same manner, with effects varying with edge age, orientation, structure, and the intervening habitat matrix (Murcia 1995). Nesting birds and their predators may therefore vary their response to different edge types. Lahti (2001) has suggested that exploring species-specific predator behaviors will be a more fruitful approach to understanding patterns in avian nest predation, given the possibility that different predator species respond to landscape features in different ways.

**Figure 1:**
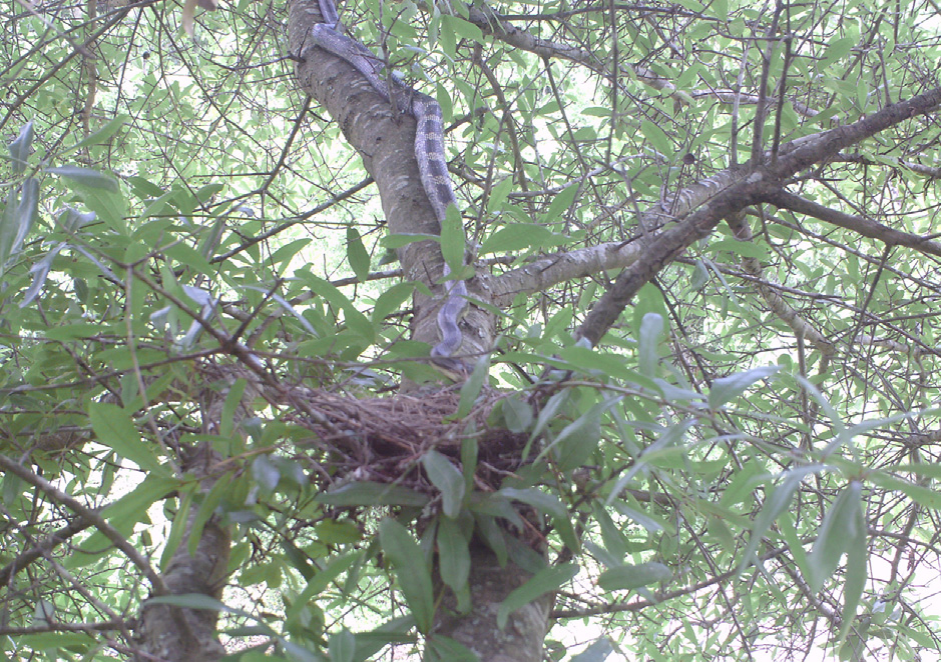
A ratsnake (*Elaphe obsoleta*), the dominant avian nest predator at our study site, has just preyed on a mourning dove (*Zenaida macroura*) nestling and is returning to the empty nest.

Increased rates of nest predation near edges has led to the study of predator autecology within edges or highly fragmented landscapes (Dijak and Thompson 2000; Chalfoun et al. 2002). Numerous nest predators, including brown-headed cowbirds (*Molothrus ater*), mammalian mesopredators, and snakes, have been shown to preferentially occupy habitat edge over interior (Gates and Evans 1998; Blouin-Demers and Weatherhead 2001a; Chalfoun et al. 2002). The mechanisms underlying predator preference of edge vary by predator group. Rat snakes (*Elaphe obsoleta;* Fig. 1), the dominant ectothermic nest predator in southeastern North America (DeGregorio et al. In Press), use edge habitat for its thermal qualities, which facilitate efficient digestion and gestation (Blouin-Demers and Weatherhead 2001b, 2002; Carfagno and Weatherhead 2006). Mammalian mesopredators use edge habitats for foraging and travel corridors (Frey and Conover 2006). Avian predators, such as raptors, corvids, and cowbirds, use edge for the increased visibility provided by perching structures adjacent to open habitats or because of the high density of passerine nests (Evans and Gates 1997; Gates and Evans 1998). Edges associated with power line right-of-ways may be especially preferred by these predator groups because edges are abrupt and well defined, vegetation below power lines is frequently managed, and the power line structures provide hunting perches for avian predators (Knight and Kawashima 1993; Rich et al. 1994; Anderson and Burgin 2008). Because each predator group uses corridors and their edges for different purposes, it is not unreasonable to assume that these different landscape features may attract different nest predators. If nest predator communities vary with landscape features, then patterns of nest predation might also vary. Understanding how predator distribution across a landscape influences nest survival has been hampered by our inability to reliably identify nest predators, which until recently was not possible.

Miniature video cameras now allow researchers to identify nest predators unambiguously (Thompson et al. 1999; Reidy and Thompson 2012; Thompson and Ribic 2012). In some cases, the most visible or abundant potential predators at a site may not actually be important nest predators (Liebezeit and Zack 2008). Thus, predator abundance at a site or within a habitat may not be indicative of actual nest predation risk. Additionally, nest cameras can provide insight into how landscape-level factors influence predator assemblages. In one study, nest predation risk from raptors increased with proximity to agriculture edges (Benson et al. 2010) and in another, decreasing forest land cover increased predation risk from cowbirds and decreased predation risk from rodents (Cox et al. 2012a). In Texas, predation by cowbirds increased with urbanization and nest height and also increased with more open land use in the landscape (Reidy and Thompson 2012). Our goal was to use nest cameras to evaluate edge-related effects, specifically those associated with power lines and unpaved roads, on predation risk from different predators at songbird nests. We test the hypothesis that proximity to power lines will decrease overall nest survival. If an increase in nest predation associated with power lines is a consequence of power lines creating edge habitat, then we expect an increase in predation with proximity to roads and forest edges. Similarly, the same suite of nest predators should be responsible for both patterns. Alternatively, if avian nest predators use power line right-of-ways because of the hunting perches provided by the poles and lines, nest predation should be higher near power lines than near roads because the former provide both edges and perches. Also, birds should be more frequent nest predators near powerlines than near roads and other forest edges.

## Materials and Methods

### Study site

**Figure 2:**
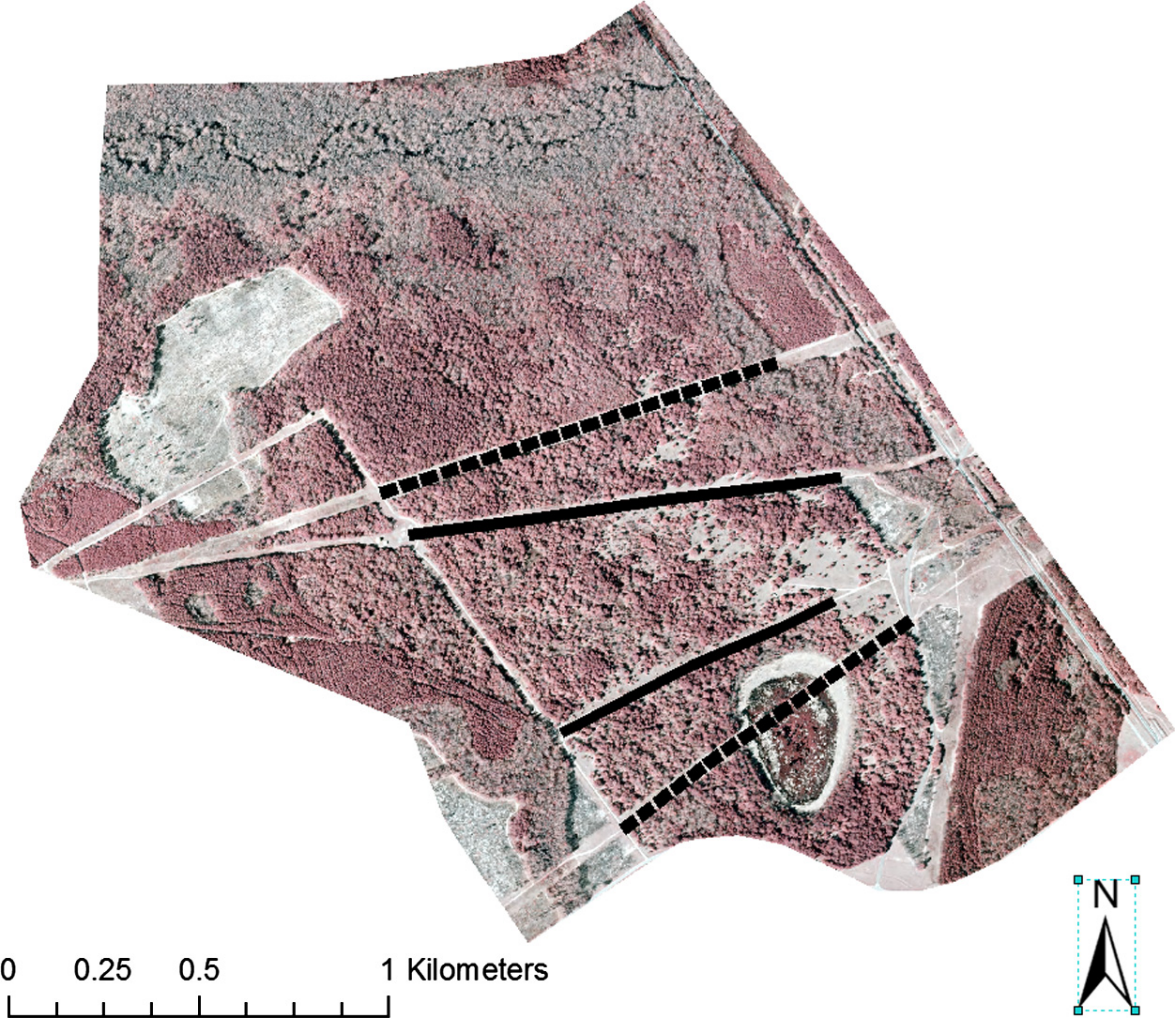
Ellenton Bay Set Aside Research Area, in Aiken County, South Carolina, is approximately 800 ha in size and is comprised of highly fragmented forest habitat. The two unpaved roads that were surveyed for predators are marked with solid black lines and the two powerline right-of-ways that were surveyed are marked with dotted black lines.

We conducted research at the Ellenton Bay Set Aside Research Area on the U.S. Department of Energy Savannah River Site in Aiken County, South Carolina. Ellenton Bay is an 800 ha area that was once row-crop agriculture and pasture but has been reverting to forest since 1951 (Fig. 2). The habitat is now mature forest intermixed with areas of open shrubland. The site is bounded to the north by a creek and floodplain forest and to the south by a two-lane paved road with daily traffic by site employees. The site is bisected by four parallel corridors running East to West, two of which are power line right-of-ways (45 m wide) and two of which are unpaved roads (30 m wide). The roads are used infrequently, primarily by field researchers. The power line corridors are maintained by South Carolina Electric and Gas Co. and are mowed at least once a year in late summer. Shrubland patches within the right-of-ways are treated with herbicides if they attain heights >4 m. Conversely, the roads are bordered by approximately 30 m of infrequently maintained shrubland habitat. Thus, edges along roads are more gradual than those along power lines.

### Daily nest survival

To assess daily songbird nest survival in relation to landscape features, we located and monitored avian nests from 5 May to 15 August 2011 and 15 March to 1 August 2012 and 2013. We monitored the nests of a variety of shrub and low-canopy nesting bird species (Appendix S1). We accumulated enough data to individually analyze daily survival rate of five species [northern cardinals (*Cardinalis cardinalis*), brown thrashers (*Toxostoma rufum*), blue grosbeaks (*Passerina caerulea*), indigo buntings (*P. cyanea*), and white-eyed vireos (*Vireo griseus*)]. The five focal species are abundant at our site and their nests could be located and monitored easily. Each of the focal species nests in all available macrohabitat types (see descriptions below) at our site. We located nests using systematic searching and behavioral cues. We filmed a subset of nests using 15 user-built miniature video systems (Cox et al. 2012a,b). Although we preferentially filmed the nests of the focal species, we filmed the nests of other species when nests of the focal species were unavailable or camera systems were unused. We placed cameras 0.5–1 m from nests and camouflaged them with nearby vegetation to reduce the likelihood of the cameras attracting predators (Richardson et al. 2009). We put cameras only on nests that were incubating or brooding to reduce the risk of nest abandonment. We checked all nests (with and without cameras) every 48 h following the protocol described by Martin and Geupel (1993). We considered a nest successful if it fledged at least one nestling or depredated if nestlings disappeared earlier than 2 days before average fledging dates for the species. At nests suspected to have fledged young, we spent considerable time (up to 2 h in two consecutive days) in the area to confirm the presence of fledglings or parents carrying food to rule out predation late in the nestling period. If fate of the nest was still uncertain, we excluded the last monitoring interval. Following predation (full or partial) of a nest with a camera, we reviewed the video the same day to identify the predator. We considered multiple visits to a nest from the same predator species as one predation event, even if they occurred on different days, because we did not know whether this was more than one individual. Similarly, if multiple predators of the same species attended a nest simultaneously (e.g., five crows simultaneously took nestlings from a northern cardinal nest), we again considered this as only one predation event. If more than one predator species removed contents from the same nest, we considered these independent events.

To assess the influence of landscape features on daily nest survival, we used logistic exposure (Shaffer 2004) with Proc GENMOD in SAS 9.2 (SAS Institute, Cary, NC). We developed models using macrohabitat type, distance to power lines, distance to road, and distance to nearest forest edge. It should be noted that distance to nearest forest edge was in some cases the same as distance to the edges of power lines or roads, although the values were not strongly correlated (linear regression: *r*^2^ < 0.02). Macrohabitat type was assessed for each nest in the field and was categorized as forest, shrubland, forest edge, pine plantation, or wetland. We defined forest edge consistent with definitions from the snake literature (e.g., Carfagno and Weatherhead 2006) and considered a nest to be in forest edge if it was less than 15 m in either direction of the interface between forest and any open habitat. The distance from each nest to the nearest road and power line was measured in the field with a tape measure if the distance was <100 m and was measured using ArcMap 10.0 (ESRI, Redlands, CA) if the distance was >100 m. We measured the distance from each nest to directly below the power line and to the nearest tire rut of a road. Distance from each nest to the nearest edge was always measured in the field because many edges were not discernible from aerial photographs. We used Akaike's Information Criterion for small sample sizes (AICc) to rank models for each analysis. We assessed models for all nesting species combined and then for each of the five focal species separately.

To assess the influence of landscape features on nest predator identity, we used a multinomial logistic regression model with Proc GLIMMIX in SAS 9.2. The data consisted of each 24-h interval a nest was filmed and the “response” of each nest at the end of the interval. Responses were predation by rat snake, corn snake (*Elaphe guttata*), racer (*Coluber constrictor*), coachwhip (*Masticophis flagellum*)*,* raptor (*Buteo* spp, *Accipiter* spp, or *Elanoides forficatus*), corvid (*Corvus brachyrhynchos, C. ossifragus*, or *Cyanocitta cristata*), brown-headed cowbird, ant (*Solenopsis invicta, Chromatagaster sp*), mammal (*Procyon lotor* or *Lynx rufus*), other avian (nonpredatory passerines or owls) or survived. Ideally, we would have assessed the response of each predator species independently. However, limited sample sizes for some predators (e.g., swallow-tailed kites [*n* = 2], blue jay [*n* = 3]) necessitated the creation of the generic groups “mammals,” “corvids,” and “raptors”, despite differences in their ecology and behavior. We excluded nests for which predator identity could not be ascertained due to camera failure and nests that failed for reasons unrelated to predation (e.g., storms). For this analysis, we filmed nests of the five focal species as well as northern mockingbirds (*Mimus polyglottos*), mourning doves (*Zenaida macroura*), yellow-breasted chats (*Icteria virens*), painted buntings (*Passerina ciris*), and eastern towhees (*Pipilo erythrophthalmus*). We evaluated support for each of the following models: macrohabitat type, distance to nearest powerline, distance to nearest unpaved road, and distance to nearest forest edge. We used Akaike's Information Criterion for small sample sizes to rank models.

### Predator behavior

In addition to monitoring songbird nests, we used radiotelemetry to track the activity and macrohabitat use of rat snakes and racers during the avian nesting seasons of 2011–2013. Both rat snakes and racers are important nest predators in our study region (Thompson and Ribic 2012; DeGregorio et al. In Press), the activity and habitat use of which have been linked to nest predation risk (Sperry et al. 2008, 2010; Klug et al. 2010; Weatherhead et al. 2010). Snakes were captured opportunistically by hand throughout the nesting season. Snakes were captured as part of a larger study investigating their spatial ecology and respective roles as nest predators. Search activities were randomly distributed across the landscape with no particular emphasis placed on roadways, so snakes were not captured disproportionately along power lines or unpaved roads. We transported snakes to a veterinarian who surgically implanted transmitters (model SI-2T 9 g, 11 g, or 13 g; Holohil Systems Ltd, Carp, ON, Canada) following Blouin-Demers and Weatherhead's (2001a) modification of Reinert and Cundall's (1982) technique. All transmitters weighed <3% of the snake's total mass. Snakes were released at their capture location 3–5 days following surgery. Snakes were tracked at various times throughout the day and night at approximately 48 h intervals and locations were recorded using GPS. At each snake location, we recorded behavior and local habitat characteristics. We plotted each snake location on an aerial photograph of the study site and used ArcMap 10.0 to measure the distance from each snake point to the nearest power line and road. We then used the buffer tool in ArcMap 10.0 to create 35 m buffers around each power line right-of-way and unpaved road, which allowed us to quantify use (number of snake locations within each buffer) and availability (proportion of study site comprised of power line or road buffers). We chose the 35 m buffer size to account for 15 m edges along either side of the corridor and an additional 5 m to span the width of the road or power line. We used analysis of variance to compare snake use of each habitat feature with 1000 random points distributed across the study site generated with ArcMap.

We also surveyed avian predators along both power line right-of-way corridors (0.81 and 1.2 km long) and unpaved roads (0.93 and 0.91 km long). Twice per month during the nesting season in 2012 and 2013 (April–July), we walked the length of each road and power line right-of-way and recorded all birds that were seen or heard. Although all birds were recorded, only potential nest predators were included in analyses. We recorded only birds that were within or on the edge of the corridor. Birds heard in the forests on either side of the survey transect were not recorded unless we determined that they were within 15 m of the forest edge. We performed all surveys in the early morning (between 0600 and 1000). We surveyed all four corridors on the same morning to standardize environmental conditions, and we varied the order in which the corridors were surveyed between days to avoid time-of-day effects. Only one author (B.A.D.) conducted surveys to eliminate interobserver variability.

We grouped birds detected as crows, raptors, cowbirds, or jays for analyses. We estimated relative density of each group for each survey (number of each predator detected/length of corridor surveyed). We then used multivariate analysis of variance with a Tukey's post hoc test to compare the mean density of predator groups between and within the two corridor types.

## Results

### Daily nest survival

**Figure 3:**
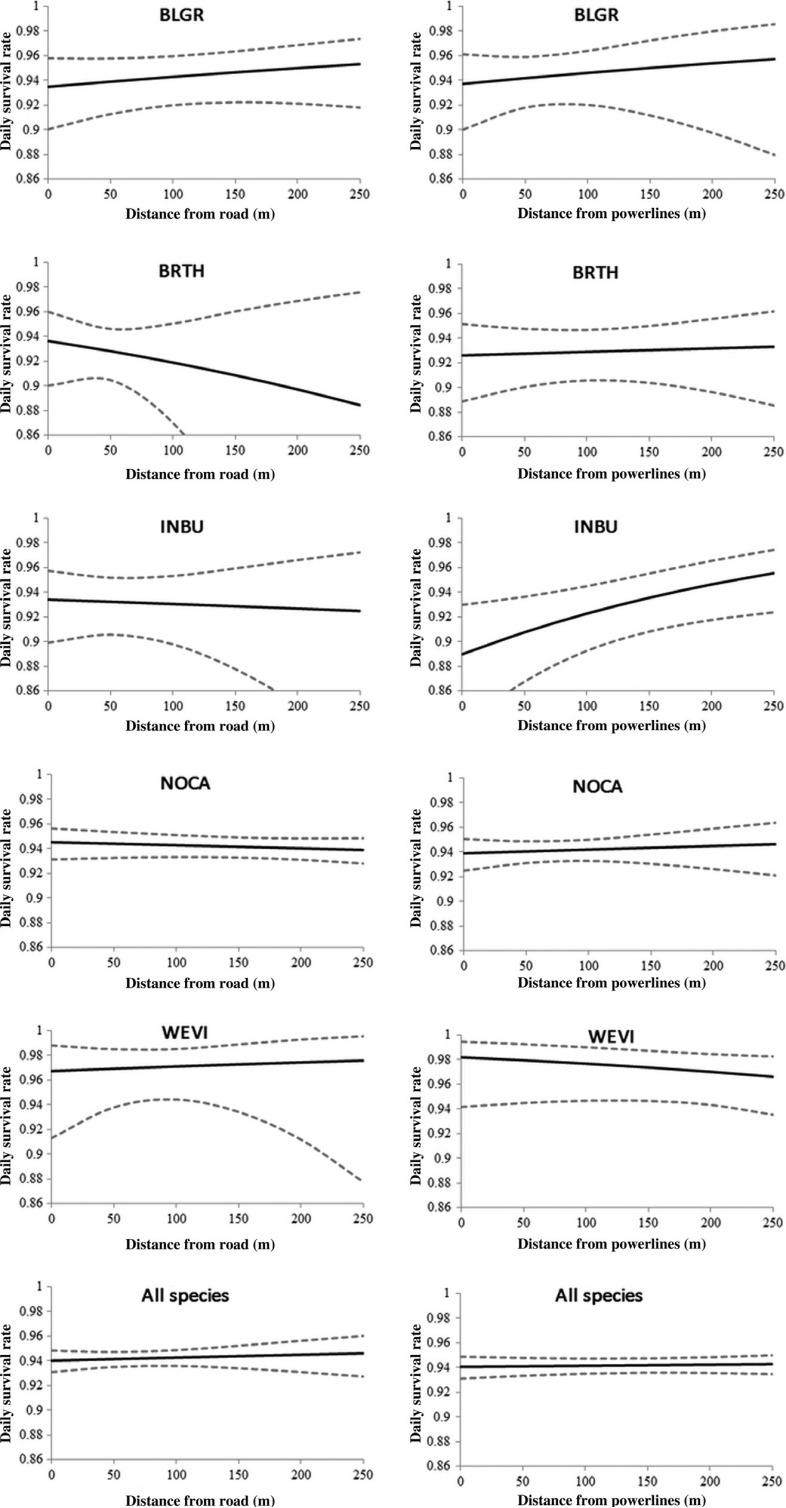
Modeled daily nest survival rates (±95% confidence intervals) for five focal songbird species as a function of distance to unpaved roads (left column) and distance to powerlines (right column).

We located and monitored 463 nests of 17 species (Appendix S1), 415 of which belonged to the five focal bird species, for a total of 5680 exposure days (focal species = 5259 exposure days). We monitored 257 northern cardinal nests (3306 exposure days), 53 brown thrasher nests (637 exposure days), 42 blue grosbeak nests (559 exposure days), 42 indigo bunting nests (448 exposure days), and 18 white-eyed vireo nests (309 exposure days). For all species combined, the top-ranked model from our set of six candidate models influencing daily nest survival rate was the distance to nearest unpaved road (Table 1). This model accounted for 78% of the total weight of evidence, and no other model was within 3.8 delta AICc units. The effect size for this model was negative and relatively mild (Fig. 3) and the next top-ranked model was constant survival accounting for 11% of the weight of evidence. When analyzed individually, distance to nearest road was not a top-ranked model for any of our five focal species. For indigo buntings, the top-ranked model from our set of five candidate models influencing daily nest survival was the distance to nearest power line (Table 1). This model accounted for 81% of the total weight of evidence, and no other model was within 4.9 delta AICc units. However, models for nearest power line were poorly supported for all other focal species. For cardinals, brown thrashers, and white-eyed vireos the top-ranked model was constant survival (Table 1) indicating that landscape factors had little influence on species-specific daily nest survival at our site, although limited sample sizes for individual species may have influenced this result. The top-ranked model for blue grosbeaks was distance to the nearest forest edge. This model accounted for 39% of the total weight of evidence but was only 1.1 delta AICc units above the next model, which was constant survival. Distance to unpaved roads was not a top-ranked model for any of our five focal species and both distance to road and distance to powerlines had relatively minor effects of daily nest survival (Fig. 3).

**Table 1:** Factors influencing daily nest survival rate of 463 nests (5680 exposure days) of shrubland-nesting birds at the Savannah River Site in South Carolina, USA during the 2011, 2012, and 2013 breeding seasons

	K	AIC	ΔAICc	wi
All species				
Distance to road	2	2401.35	0	0.78
Constant survival	1	2405.17	3.82	0.11
Distance to edge	2	2406.59	5.24	0.06
Distance to power lines	2	2407.04	5.69	0.05
Macrohabitat type	5	2411.51	10.16	0.00
Species	16	2424.29	25.86	0.00
BLGR				
Distance to edge	2	201.684	0	0.48
Constant survival	1	207.16	1.19	0.26
Distance to power lines	2	208.28	2.59	0.13
Distance to road	2	209.26	3.15	0.10
Macrohabitat type	5	212.60	6.48	0.02
BRTH				
Constant survival	1	273.10	0	0.37
Distance to edge	2	274.11	1.01	0.22
Distance to road	2	274.90	1.80	0.15
Macrohabitat type	4	175.12	2.02	0.13
Distance to power lines	2	275.229	2.13	0.13
INBU				
Distance to power lines	2	188.55	0	0.83
Constant survival	1	193.48	4.93	0.07
Macrohabitat type	5	194.29	5.74	0.05
Distance to edge	2	195.51	6.96	0.03
Distance to road	2	195.71	7.16	0.02
NOCA				
Constant survival	1	1225.21	0	0.44
Distance to power lines	2	1226.68	1.47	0.21
Distance to road	2	1227.02	1.81	0.18
Distance to edge	2	1227.24	2.03	0.16
Macrohabitat type	5	1232.03	6.81	0.01
WEVI				
Constant survival	1	76.03	0	0.59
Distance to power lines	2	78.33	2.30	0.19
Distance to edge	2	79.38	3.34	0.11
Distance to road	2	79.39	3.36	0.11
Macrohabitat type	4	89.69	13.65	0.00

### Predator identification

We deployed nest cameras at 206 nests and confirmed predator identity for 137 predation events (Table 2). This includes 10 occasions in which more than one predator preyed on the same nest. Twelve nests failed due to nonpredation events or were abandoned and 67 nests successfully fledged at least one bird without any documented predation of eggs or nestlings. Snakes collectively were the most frequent nest predators, accounting for 80 predation events. Rat snakes were the most frequently documented snake species (38 events), followed by corn snakes (20 events), black racers (17 events), and coachwhips (five events). Avian predators were the next most frequent group, with 40 predation events attributed to at least nine species. Of the avian predators, crows (American and fish) and blue jays were responsible for 14 predation events and brown-headed cowbirds for 11 events. Ants and mammals were responsible for eight and nine predation events, respectively. We attributed predation at six nests to avian predators that did not fit into the previous avian predator groups (owls or non-cowbird passerines).

**Table 2:** Nest predators identified using miniature video cameras at 206 songbird nests at the Savannah River Site in South Carolina, USA from 2011 to 2013. We recorded a total of 137 nest predation events, in 10 instances multiple predators preyed on the same nest

Nest predator or fate		2011	2012	2013
Snakes		13	31	36
Rat snake	*Elaphe obsoleta*	5	19	14
Corn snake	*Elaphe guttata*	3	8	9
Black racer	*Coluber constrictor*	4	3	10
Coachwhip	*Masticophis flagellum*	1	1	3
Avian predators		12	12	16
Brown-headed cowbird	*Molothrus ater*	4	3	4
Crows	*Corvus brachyrhnchos* or *C. ossifragus*	3	2	6
Blue jay	*Cyanocitta cristata*	1	1	1
Swallow-tailed kite	*Elanoides forficatus*	2	0	0
Buteo	*Buteo spp*.	1	2	1
Accipter	*Accipter spp*.	0	2	1
Eastern screech owl	*Otus asio*	1	2	0
Carolina wren	*Thyothorus lutovicianus*	0	0	1
Gray catbird	*Dumetella carolinensis*	0	0	2
Ants	*Chromatagaster spp. Solenopsis spp*.	2	3	3
	*Solenopsis invicta*			
Mammals		2	3	4
Raccoon	*Procyon lotor*	2	2	4
Bobcat	*Felis rufus*	0	1	0
Unknown fate (camera malfunction, not visible)		1	6	5
Fledged nests		17	20	30

**Figure 4:**
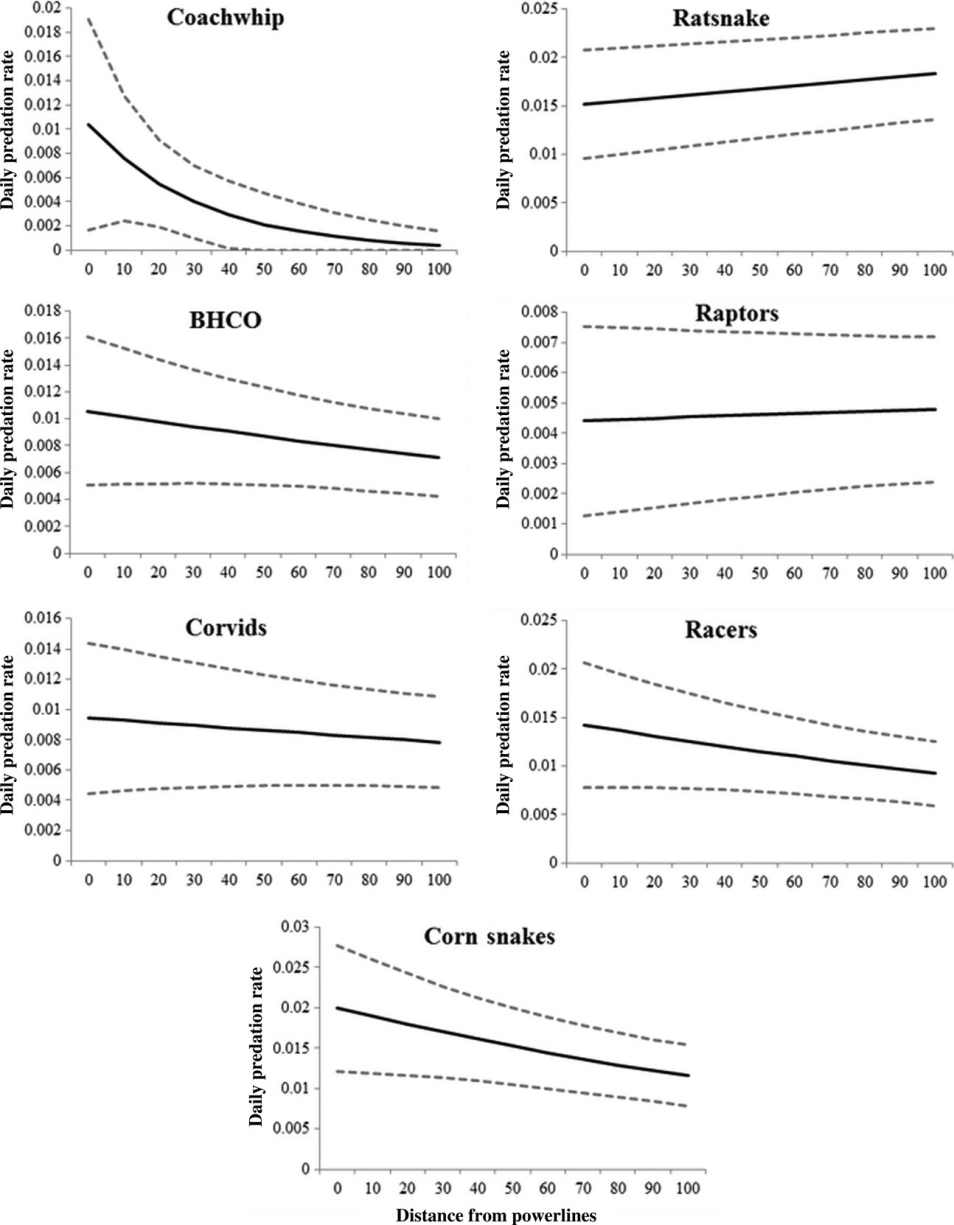
Daily nest predation rate (±85% confidence intervals) by different nest predators as a function of their distance from powerlines.

Distance to power lines was the best predictor of predator identity. This model accounted for 96% of the weight of evidence and all other models were at ≥8 delta AICc units below it (Table 3). Daily predation risk from coachwhips was most influenced by distance to power lines, with the odds of a nest being preyed on by coachwhips relative to surviving decreasing by 1.4% for each 10 m increase in distance from power lines (*β* = −0.032; 85% CI: −0.036, −0.028). The odds of predation by black racers (*β* = −0.0046; 85% CI: −0.0051, −0.0041), corn snakes (*β* = −0.0057; 85% CI: −0.0062, −0.0052), brown-headed cowbirds (*β* = −0.0042; 85% CI: −0.0048, −0.0037), and corvids (*β* = −0.0021; 85% CI: −0.0026, −0.0016) each decreased by approximately 1% for each 10 m moved away from power lines (Fig. 4). Predation risk from ants (*β* = −0.00047; 85% CI: −0.00096, −0.0002) and mammals (*β* = −0.0006; 85% CI: −0.00059, −0.00046) also increased with proximity to power lines, but these effects were weak and had confidence intervals that encompassed zero, indicating uncertainty. Contrary to our predictions, predation by rat snakes (*β* = 0.0016; 85% CI: 0.0014, 0.0019) and raptors (*β* = 0.00059; 85% CI: 0.0008, 0.0010) decreased by approximately 1% with each 10 m nearer to power lines. Although the models received little support, predation by rat snakes was positively associated with distance to nearest forest edge (*β* = −0.0016; 85% CI: −0.0024, −0.0008) and even more strongly with nearest unpaved road (*β* = −0.0028; 85% CI: −0.0034, −0.0022). Predation by raptors was also positively associated with distance to nearest road (*β* = −0.0043; 85% CI: −0.0056, −0.0031) but negatively associated with distance to nearest forest edge (*β* = 0.0050; 85% CI: 0.0038, 0.0062).

**Table 3:** Influence of landscape features on nest predator identity for 198 bird nests filmed from 2011 to 2013 at the Ellenton Bay Set Aside Research Area

	−2logL	AIC	ΔAICc	wi
Distance to power lines	1585.31	1625.77	0	0.96
Distance to edge	1593.73	1634.19	8.42	0.02
Distance to road	1599.71	1640.17	14.4	0.01
Macrohabitat type	1547.24	1650.08	24.31	<0.01

### Predator behavior

From May–August 2011 and March–August 2012 and 2013, we used radiotelemetry to track 33 rat snakes and 16 black racers accumulating 1387 and 755 locations, respectively. Power lines and unpaved roads comprised 5% and 7% of the study site, respectively. Snake use of power lines was nonrandom and also differed by species: racers were found near power lines at 17% of relocations and rat snakes only 2% of relocations (*F* = 10.85, *P* = 0.01). Use of roads by both species exceeded that expected by chance, with racers using roads at 19% of relocations and rat snakes at 10% of relocations (*F* = 14.48, *P* = 0.008). Thus, both snake species were positively associated with roads but exhibited opposite responses to power lines.

**Figure 5:**
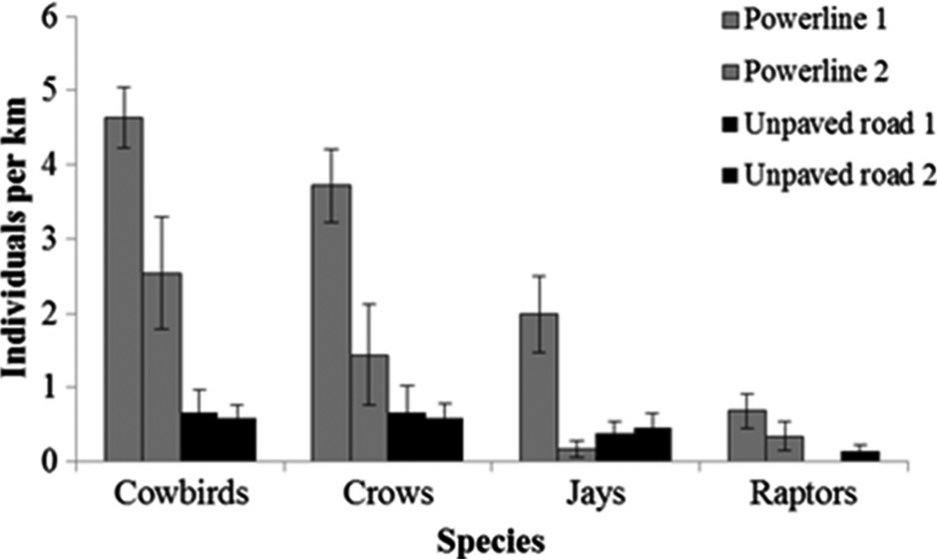
Mean density (±SE) of avian nest predators detected along two powerline right-of-way corridors and two unpaved road corridors at the Savannah River Site, South Carolina, USA. Each corridor was surveyed 13 times during the avian nesting seasons of 2012 and 2013.

We surveyed each unpaved road and each power line 13 times during the 2012 and 2013 breeding seasons. We detected a total of 102 brown-headed cowbirds, 78 crows (American and fish), 37 blue jays, and 14 raptors (red-tailed hawks [*Buteo jamaicensis*], American kestrels [*Falco sparverius*], and Mississippi kites [*Ictinia mississippiensis*]). Overall, predator density differed between power lines and unpaved roads (*F*_4,47_ = 11.01, *P* = 0.001). Brown-headed cowbirds (*F* = 35.35, *P* = 0.001), crows (*F* = 14.73, *P* = 0.001), and raptors (*F* = 7.80, *P* = 0.007) were more dense along power lines than roads (Fig. 5). There was some evidence that blue jays were also more abundant along power lines (*F* = 5.74, *P* = 0.057). There were differences in density between the two surveyed power lines with more cowbirds, crows, and jays detected at power line right-of-way 1 relative to 2 (*P* > 0.01). However, power line 2 still had greater densities of cowbirds compared with either of the unpaved roads (*P* < 0.03). Power line 1 had greater densities of blue jays than power line 2 (*P* = 0.01) and than either of the unpaved roads (*P* < 0.03). There was no difference in raptor density between the two power lines surveyed (*P* = 0.02). No significant differences in predator density were detected between the two unpaved roads (*P* > 0.98).

## Discussion

Three general patterns emerged from our results. First, distance to nearest power line strongly influenced nest predator identity at our site. Predators that used power lines and poles as perching structures (crows and cowbirds) or that used the frequently maintained shrub habitat under power lines (coachwhips and racers) frequently preyed on nests near power lines. Second, distance to power lines had relatively little impact on daily nest survival for 4 of the 5 focal songbird species. Only indigo bunting daily nest survival was strongly influenced by distance to nearest power line (Fig. 3). We interpret this as a consequence of the relative importance of each predator to overall nest predation. Similar with results from other studies of nest predators (Thompson et al. 1999; Weatherhead et al. 2010), rat snakes were more important nest predators than racers. In fact, rat snakes were the locally dominant nest predator at our site, accounting for 28% (38 of 137 predation events) of all filmed predation. Although proximity to power lines was a strong predictor of nest vulnerability to some predators, the opposite was true for rat snakes and raptors. Third, different predators used landscape features differently. Radiotelemetry revealed that rat snakes rarely used power line right-of-ways but were often associated with unpaved road edges. Raptors, corvids, and cowbirds were more frequently encountered along power line right-of-ways than along unpaved road corridors. In some cases, these distribution patterns were reliable predictors of nest predator identity, but in others (raptors), they were misleading.

Rat snakes located with radiotelemetry were disproportionately near unpaved roads. The association of rat snakes with unpaved roads was actually an association with the adjacent edge habitat (only once was a ratsnake encountered on a road). Edge use by rat snakes has been well documented (e.g., Blouin-Demers and Weatherhead 2001a,b; Carfagno and Weatherhead 2006). Contrary to our predictions, rat snakes rarely used the edges associated with power lines, suggesting that not all edges are the same from a snake's perspective. We suggest that because edges associated with power lines are abrupt they do not provide the thermal heterogeneity for which rat snakes use edges. In the only study to date that reported snake use of different edge types, Blouin-Demers and Weatherhead (2001a) found that rat snakes used both natural and artificial edges equally. Artificial edges in that case were predominantly the interface between field and forest habitats, which may be less abrupt than edges associated with power lines. Alternatively, that study took place in Ontario where rat snakes are more thermally challenged (Blouin-Demers & Weatherhead 2001c) than those in South Carolina, potentially increasing the reliance of snakes on edges regardless of structure. Edges along unpaved roads at our site were gradual and may have been more attractive to rat snakes seeking thermally heterogeneous habitat. Unlike rat snakes, racers at our site often used power line right-of-ways. We suggest that racers, a grassland and shrubland species (Plummer and Congdon 1994; Keller and Heske 2000), were using power lines for the early-successional habitat associated with power lines due to their frequent mowing. It is also possible that rat snakes avoid power line right-of-ways because they can be preyed on by the raptors that use the poles as perches. However, it is unclear why rat snakes would avoid predation associated with these landscape features whereas racers, coachwhips, and corn snakes were often located near power lines and preyed on nests under power lines. Future investigations of edge use by snakes should quantify the properties and use of different types of edges, but our results make it clear that even at the same site, different types of edges can have different ecological effects.

Proximity to unpaved roads was the best-supported model for influencing daily nest survival rate. The effect of distance to nearest unpaved road on daily survival rate was negative but mild. Although rat snakes were the most frequently documented nest predator at our site, we also filmed at least 16 additional species of nest predators. Unpaved roads affected nest survival when all nesting species were combined for analyses, yet when nesting species were analyzed separately, distance to unpaved roads was never included in the top models. We suggest that this is a consequence of smaller sample sizes for individual species.

We predicted that the density of potential avian nest predators would be higher near power lines because they use the associated perching structures and therefore that predation of nests near power lines would more often be attributed to avian predators. Raptors and corvids are thought to associate with power lines because they use them for hunting perches and nest sites (Knight and Kawashima 1993). Similarly, cowbirds are often abundant near power lines because they forage in the mowed grass beneath them (Rich et al. 1994) and perch on the lines to watch for nests to parasitize (Evans and Gates 1997; Gates and Evans 1998). We did find that raptors, crows, and cowbirds occurred in higher densities near power lines relative to unpaved road corridors and that greater abundance of cowbirds and corvids near power lines did increase predation risk from these predators at our site. However, greater predator abundance in an area does not necessarily translate to higher nest predation from that predator (Liebezeit and Zack 2008), which was the case for raptors. Although we often observed red-tailed hawks using power lines, they were observed as frequently preying on nests as Accipiters, a forest interior-associated group of raptors. This discrepancy is due to species-specific responses. Although raptors were positively associated with power lines, they were more likely to prey on nests away from power lines. Red-tailed hawks, while abundant near power lines, may be using perches to hunt for mammalian prey rather than avian nests. Without nest cameras, we may have erroneously concluded that raptors were more frequent predators of nests near power lines. Responses of predators to landscape features will be species-specific and grouping predators in broad taxonomic groups (e.g., “raptors”) might mask trends. Examining species-specific patterns in predation will require large sample sizes of predation events, which can be logistically infeasible to acquire for infrequent nest predators. Additionally, density of potential avian nest predators may vary between power lines. For instance, only one of the power line right-of-ways that we surveyed had higher densities of blue jays than unpaved roads. Also, density of crows and cowbirds was greater at one power line than the other. However, even the power line with lower predator density still had higher densities of crows and cowbirds than either of the unpaved roads.

Our results indicate that landscape features can affect daily nest survival because of their influence on nest predator distribution and behavior. Predator response to landscape features is likely to be species-specific and influenced by geographic location and the surrounding habitat matrix, confounding our ability to make broad generalizations. Whereas both unpaved roads and power line right-of-ways fragment forests and create linear edge habitat, they are used differently by predators. Numerous predators at our site were associated with power line right-of-ways (e.g., racers, cowbirds, raptors), but their relative importance as nest predators at this site was minor relative to rat snakes. For many shrubland-nesting species in the Northeastern United States, power line right-of-ways provide refuges of shrub habitat (Kubel and Yahner 2008; King et al. 2009). The importance of nest predators varies geographically (Thompson and Ribic 2012). In areas where shrubland habitat is restricted to power line right-of-ways, these habitats could act as ecological traps by increasing encounters between power line-associated nest predators and imperiled bird species. Our results suggest that nest predator identity can be influenced by landscape features, although this may not necessarily drive trends in nest survival. Broad generalizations about the influence of landscape features such as unpaved roads and power lines, are likely to be region-specific and driven by the behavior and identity of local nest predators. Further work investigating the geographic trends in importance of nest predators (Thompson and Ribic 2012; DeGregorio et al. In Press) and the behavior of these nest predators in relation to landscape features will be necessary to understand the mechanisms influencing avian nest survival in relation to landscape features.

## Supporting Information

**Appendix S1.** Nests that were monitored at Ellenton Bay Set Aside Research Area during the 2011, 2012, and 2013 nesting seasons.
