# Supplementary material for: Power lines, roads, and avian nest survival: effects on predator identity and predation intensity
Source: Ecol Evol. 2014 Mar 31;4(9):1589–600. doi: 10.1002/ece3.1049 (PMC4063460; doi:10.1002/ece3.1049)
Supplement: Supplementary file 1 [file ece30004-1589-SD1.docx]

Appendix A1: Nests that were monitored at Ellenton Bay Set Aside Research Area during the

2011, 2012, and 2013 nesting seasons.

| Species |  | Number of Nests |
| --- | --- | --- |
| Blue Grosbeak | Passerina caerulea | 42 |
| Brown Thrasher | Toxostoma rufum | 53 |
| Carolina Wren | Thryothorus ludovicianus | 3 |
| Common Yellowthroat | Geothlypis trichas | 1 |
| Eastern Towhee | Pipilo erythrophthalmus | 3 |
| Indigo Bunting | Passerina cyanea | 42 |
| Mourning Dove | Zenaida macroura | 21 |
| Northern Cardinal | Cardinalis cardinalis | 260 |
| Northern Mockingbird | Mimus polyglottos | 7 |
| Northern Parula | Parula americana | 2 |
| Orchard Oriole | Icterus spurius | 2 |
| Painted Bunting | Passerina ciris | 11 |
| Prairie Warbler | Dendroica discolor | 2 |
| Red-eyed Vireo | Vireo olivaceus | 1 |
| Red-winged Blackbird | Agelaius phoeniceus | 1 |
| Yellow-billed Cuckoo | Coccyzus americanus | 7 |
| Yellow-breasted Chat | Icteria virens | 2 |
